# Linkage analysis using whole exome sequencing data implicates *SLC17A1, SLC17A3, TATDN2* and *TMEM131L* in type 1 diabetes in Kuwaiti families

**DOI:** 10.1038/s41598-023-42255-2

**Published:** 2023-09-11

**Authors:** Prashantha Hebbar, Rasheeba Nizam, Sumi Elsa John, Dinu Antony, Mohammad Dashti, Arshad Channanath, Azza Shaltout, Hessa Al-Khandari, Heikki A. Koistinen, Jaakko Tuomilehto, Osama Alsmadi, Thangavel Alphonse Thanaraj, Fahd Al-Mulla

**Affiliations:** 1 Department of Genetics and Bioinformatics, Dasman Diabetes Institute, 15462 Kuwait City, Kuwait; 2 Department of Population Health, Dasman Diabetes Institute, Kuwait City, Kuwait; 3 Department of Pediatrics, Farwaniya Hospital, Ministry of Health, Kuwait City, Kuwait; 4 Department of Medicine, University of Helsinki and Helsinki University Hospital, Helsinki, Finland; 5 Department of Public Health and Welfare, Finnish Institute for Health and Welfare, Helsinki, Finland; 6 Minerva Foundation Institute for Medical Research, Helsinki, Finland; 7 Department of Public Health, University of Helsinki, Helsinki, Finland; 8 Diabetes Research Group, King Abdulaziz University, Jeddah, Saudi Arabia; 9 King Hussein Cancer Center, Amman, Jordan

**Keywords:** Genetics, Genetic linkage study, Genetic markers, Quantitative trait, Sequencing

## Abstract

Type 1 diabetes (T1D) is characterized by the progressive destruction of pancreatic β-cells, leading to insulin deficiency and lifelong dependency on exogenous insulin. Higher estimates of heritability rates in monozygotic twins, followed by dizygotic twins and sib-pairs, indicate the role of genetics in the pathogenesis of T1D. The incidence and prevalence of T1D are alarmingly high in Kuwait. Consanguineous marriages account for 50–70% of all marriages in Kuwait, leading to an excessive burden of recessive allele enrichment and clustering of familial disorders. Thus, genetic studies from this Arab region are expected to lead to the identification of novel gene loci for T1D. In this study, we performed linkage analyses to identify the recurrent genetic variants segregating in high-risk Kuwaiti families with T1D. We studied 18 unrelated Kuwaiti native T1D families using whole exome sequencing data from 86 individuals, of whom 37 were diagnosed with T1D. The study identified three potential loci with a LOD score of ≥ 3, spanning across four candidate genes, namely *SLC17A1* (rs1165196:pT269I), *SLC17A3* (rs942379: p.S370S)*, TATDN2* (rs394558:p.V256I), and *TMEM131L* (rs6848033:p.R190R). Upon examination of missense variants from these genes in the familial T1D dataset, we observed a significantly increased enrichment of the genotype homozygous for the minor allele at *SLC17A3* rs56027330_p.G279R accounting for 16.2% in affected children from 6 unrelated Kuwaiti T1D families compared to 1000 genomes Phase 3 data (0.9%). Data from the NephQTL database revealed that the rs1165196, rs942379, rs394558, and rs56027330 SNPs exhibited genotype-based differential expression in either glomerular or tubular tissues. Data from the GTEx database revealed rs942379 and rs394558 as QTL variants altering the expression of *TRIM38* and *IRAK2* respectively. Global genome-wide association studies indicated that *SLC17A1* rs1165196 and other variants from *SLC17A3* are associated with uric acid concentrations and gout. Further evidence from the T1D Knowledge portal supported the role of shortlisted variants in T1D pathogenesis and urate metabolism. Our study suggests the involvement of *SLC17A1*, *SLC17A3*, *TATDN2,* and *TMEM131L* genes in familial T1D in Kuwait. An enrichment selection of genotype homozygous for the minor allele is observed at *SLC17A3* rs56027330_p.G279R variant in affected members of Kuwaiti T1D families. Future studies may focus on replicating the findings in a larger T1D cohort and delineate the mechanistic details of the impact of these novel candidate genes on the pathophysiology of T1D.

## Introduction

Type 1 diabetes (T1D) is the most prevalent type of diabetes in children and adolescents, accounting for 5–10% of total diabetes cases globally^1^. The International Diabetes Federation Diabetes Atlas, 10th edition reports a global estimate of 2.61 billion cases of T1D among children and adolescents under the age of 19 years^2^. T1D is a chronic condition characterized by the progressive destruction of pancreatic β-cells, leading to an absolute deficiency of insulin and, subsequently, a lifelong dependency on treatment with exogenous insulin. Inflammation is believed to be a critical hallmark of the disease, as evidenced by the presence of multiple autoantibodies against islet cell antigens^3^.

The inherent risk of developing T1D involves genetic and environmental factors that vary across different geographical locations. Most of the patients with T1D lack a positive family history of the disease. However, an estimated heritability rate of > 50% in monozygotic twins, 6% in dizygotic twins, and 6–7% in sib-pairs indicate that genetic predisposition plays a role in the pathogenesis of this disease^4^. More than 60 genetic loci have been associated with T1D in multiple ethnic populations^5^. The heritable risk of T1D has been primarily attributed to the human leucocyte antigen (HLA) region that maps to the short arm of chromosome 6^5^. The HLA region, with extreme polymorphisms at its multiple genes, remains by far the greatest contributor to the genetic susceptibility to T1D. Several high-risk HLA DR–DQ haplotypes are useful in the diagnosis of the disease, as around 90% of patients with T1D either carry the HLA DR4-DQ8 or DR3-DQ2 haplotype, while 30% tend to carry both haplotypes^6^. An increased risk of islet autoimmunity has been reported among siblings who share the DR3/4-DQ8 haplotype with their diabetic proband^7^.

Global studies on genome-wide association and linkage analyses have revealed around 50 independent non-HLA markers associated with T1D^8–11^. A recent trans-ancestral fine mapping study implicated 78 genome wide regions including 36 novel regions linked to T1D^11^. To date, the most strongly associated T1D marker is the polymorphism of a variable number of tandem repeats in the 5’ UTR of the insulin (*INS*) gene that maps to chromosome 11p15.5^12^. Polymorphisms in other candidate genes, such as cytotoxic T-lymphocyte antigen (*CTLA4*), protein tyrosine phosphatase non-receptor type 22 (*PTPN22*), interleukin 2 receptor subunit alpha (*IL2RA*), and interferon-induced helicase (*IF1H1*), have also been widely associated with T1D^9,13–16^. Experimental studies have also suggested environmental and lifestyle-related factors such as low vitamin D, early introduction to cow’s milk, exposure to toxins, and deregulation of gut microbiota, in the pathogenesis of T1D^17^, but their causal role has not been proven. Environmental factors tend to modulate epigenetic events, including DNA methylation and histone modifications, influencing the transcriptomic profiles of key genes involved in T1D^18,19^.

Childhood diabetes broadly comprises of T1D, early onset type 2 diabetes (T2D) and other monogenic forms such as maturity onset diabetes of the young (MODY), neonatal and syndromic diabetes^20^. T1D is the most frequent form of diabetes in children and young adolescents. Most genetic association studies in T1D have been performed on Caucasians^21^. Currently, no such data have been published from Kuwait, even though the incidence and prevalence of T1D are alarmingly high in this region. As per the IDF (2021) ranking, Kuwait stands third globally, with a high incidence rate of 41.7 per 100,000 population per annum of T1D in children (IDF Atlas 10th Edition 2021)^2^. There was a surge in the incidence of T1D in children under the age of 14 years, from 17.7 per 100,000 population per year in 1992–1994 to 40.9 in 2011–2013^22^. An annual increase of 4.1% is reported in the incidence of T1D among young Kuwaiti children^22^. Nearly one third of the children diagnosed with T1D from the region presents diabetic ketoacidosis^23^.

Incidence of T2D was reported to be 2.56 per 100,000 Kuwaiti children under 14 years of age, between 2011 and 2013^24^. Prevalence of obesity in children was observed to be exceedingly high in Kuwait (30.5%) which could be the driving factor for diabetes epidemic and its associated complications^25^. A recent study by Al-Kandari et al. revealed that pathogenic mutations from known MODY genes accounted for 21.8% of the Kuwaiti MODY families investigated^26^. Diagnosis of MODY is challenging and it is often misdiagnosed as T1D due to its early onset. Globally, mutations in 14 known MODY genes account for 1–6% of diabetes in children^27^. Neonatal and syndromic diabetes are rare forms of monogenic diabetes. Neonatal diabetes is diagnosed in children under 6 months of age; mutations in 22 distinct genes have been associated with the disease^28^. Syndromic forms of diabetes are characterized by young onset age and additional non-autoimmune extra pancreatic features. Data on prevalence of MODY, neonatal and other syndromic forms of diabetes are limited from the Gulf region. Consanguineous marriages are common in Kuwait, leading to an excessive burden on the enrichment of recessive alleles and clustering of familial disorders^29^. The unique genetic profiles shaped by inbreeding and extreme climatic conditions of Kuwait further contribute to the disease development^30^.

Very limited studies have explored the link between genetic polymorphisms and susceptibility to T1D in Kuwaiti population. A significantly increased frequency of HLA DQA1*0301/DQB1*0201 haplotype is reported in Kuwaiti T1D cases compared to controls^31^. Increased occurrence of non-aspartic acid residue at position 57 of HLA *DQB1* has been reported in Kuwaiti T1D children. A previous study on vitamin D receptor polymorphisms has shown significant association of *Fok*I and *Taq*I with susceptibility to T1D in Kuwaiti children^32^. Variations in interleukin 4 and interleukin 13 genes are additionally associated with susceptibility to T1D and their co-inheritance with high-risk HLA haplotypes are reported in Kuwaiti children^33^. Our recent study has revealed differential methylation profile of islet cell autoantigen 1, a prominent marker for beta cell autoimmunity in Kuwaiti T1D families^19^. Additionally, the study revealed 84 markers differentially methylated CpG sites in T1D and validated 18 CpG using publicly available gene expression data.

To further address this knowledge gap in the increased incidence and genetic etiology of T1D among Kuwaitis, we aimed at identifying recurrent genetic variants segregating in high-risk Kuwaiti T1D families by performing linkage analyses. We hypothesized that the presented family-based study will enable us to detect population-specific genetic markers that may positively impact evidence-based national risk assessment strategies.

## Methods

### Clinical recruitment of T1D families

Families with one or more T1D cases indicating either a vertical or a horizontal transmission of the disease were selected for the study (Supplementary Fig. S1). Data and samples used in this study were obtained from the registry of the Childhood-Onset Diabetes eRegistry (CODeR), which is a comprehensive and prospective pediatric population-based diabetes registry^22^ maintained by Dasman Diabetes Institute, in collaboration with the Ministry of Health (MOH) of Kuwait. The CODeR was established in 2011 for the surveillance of children and adolescents diagnosed with diabetes in Kuwait. Newly diagnosed cases of T1D are registered electronically or manually in the national registry from hospitals across the country. Physicians report patients from a primary care (local government and private hospitals) and secondary source (Kuwait Diabetes Society, MOH primary care centers, and the clinics at Dasman Diabetes Institute). Data were extracted using standard registry forms and by individually reviewing the medical records of patients registered as having T1D. Patients referred by the registry or clinicians were contacted by the study team for enrollment in the study. After providing counselling for the proband and their first-degree relatives, blood samples were collected at the outpatient department of Dasman Diabetes Institute. This was done under the supervision of a pediatric endocrinologist who examined the participants and extracted data from their clinical records. A total of 18 Kuwaiti T1D families consisting of 37 affected probands and 49 unaffected first-degree relatives available to us were considered for inclusion in the study cohort.

Research protocols were approved by the Ethical Review Committee of Dasman Diabetes Institute and the study was performed in accordance with the principles of the Declaration of Helsinki, as revised in 2008. Written informed consent was obtained from all study participants. In the case of children, informed consent was obtained from the parents/legal guardians, and an assent was obtained from children aged seven years or more.

The diagnosis of T1D was established according to the World Health Organization (WHO) criteria, which included fasting hyperglycemia and absolute insulin deficiency, as measured by a deficient C-peptide concentration (< 0.3 nmol/l)^34^. The date of the first insulin injection was taken as the date of T1D onset. The diagnosis of T1D in children was further confirmed by measuring the GAD-antibodies. The collected data included age, gender, body mass index (BMI), nationality, date of birth, date of diagnosis, family history of diabetes in first-degree relatives, and measurements of glycated hemoglobin A1c (HbA1C), plasma glucose, blood pressure, uric acid, blood urea nitrogen, and creatinine concentrations. Inclusion criteria included (i) Families with more than one T1D cases exhibiting either a vertical or a horizontal transmission of the disease, (ii) Diagnosis of T1D established based on World Health Organization (WHO) criteria, (iii) Diagnosis of T1D was confirmed by the presence of one or more autoantibodies against pancreatic islet cells, and (iv) T1D cases of Kuwaiti-Arab origin/Ethnicity. Exclusion criteria included presence of other chronic systemic, genetic or metabolic diseases.

### Blood sample collection and processing

Blood samples were collected in ethylenediaminetetraacetic acid (EDTA)-treated tubes. Genomic DNA was extracted using a QIAamp Blood DNA kit (Qiagen, Germany) and was quantified using a Qubit Fluorometer (Thermofisher, USA), following the manufacturer’s protocol.

### Whole exome sequencing and variant calling

Exome libraries were generated at 40–50 × coverage using Nextera Rapid Capture Exome kit (Illumina Inc. USA) following the manufacturer’s protocol (Supplementary Fig. S1). A total of 50 ng of purified genomic DNA was simultaneously fragmented and tagged enzymatically. The purified fragments were ligated to adapters specific for the Illumina platform using a 10-cycle polymerase chain reaction (PCR). Libraries were denatured into single-stranded DNA and biotin labeled probes specific to the targeted region were used for the rapid capture hybridization. The pool was enriched for the targeted regions by using streptavidin beads. The enriched DNA fragments were hybridized for a second rapid capture. The captured libraries were amplified by polymerase chain reaction and quantified using a high sensitivity kit on a qubit fluorometer (Thermofisher Scientific, Massachusetts, United States). The quality of the prepared libraries was tested using Bioanalyzer (Agilent, California, United States). Enriched libraries were clustered using TruSeq Paired Cluster Kit V3 (Illumina Inc. USA) and paired end sequencing was carried out in HiSeq 2500 using Illumina’s Sequence by Synthesis technology as 100 paired end read. Pedigrees of the sequenced individuals are given in Supplementary Fig. S2.

Raw sequencing data in a BCL format was converted to Fastq data using bcl2fastq v.2.20. Quality control of Fastq files was performed using FastQC (v0.11.9). Sequence reads were aligned to the reference human genome build hg19 using BWA v.0.7.17. Marking the read pairs that are likely to have originated from duplicates of the same original DNA fragments and recalibrating base quality scores were performed on the initial BAM file. Variants were called from the processed alignment files adopting GATK’s best practice guidelines and using standard hard filtering parameters for variant discovery. GATK v.3.7 HaplotypeCaller was executed for each sample to generate an intermediate genomic variant call file (gVCF). Joint variant calling was performed on all samples using the GenotypeGVCFs tool.

### Linkage analysis of the T1D pedigrees

Prior to performing the linkage analysis, we filtered the merged VCF files for only biallelic single nucleotide polymorphisms (SNPs), and we removed low-quality SNPs using a series of filters such as percentage genotyped (> 10%), missingness per individual (> 10%), variants departing from Hardy–Weinberg Equilibrium (HWE) (> 1e-06), and Mendelian errors (families with > 10% errors and SNPs with more than 10% errors) using PLINK1.9. Variations that passed through quality control checks, including tests for Hardy–Weinberg Equilibrium and Mendelian errors were used for subsequent analyses. Analysis was restricted to variants with a minor allele frequency of ≥ 1% and a call rate of ≥ 90%. Pedigrees were built based on individuals’ pairwise genetic distances in the SNP data. KINGS software was used to test pairwise genetic relatedness between the families^35^. We retained 18 families in the final analysis based on the criteria that there was no genetic relatedness between any 2 families. Further, we converted the VCF files to the PED format and set centimorgan positions for all variants in every autosome using recombination map files of GRCh37 build using PLINK 1.9.

Parametric two-point linkage analysis between a disease trait and a set of genotyped markers, based on a recessive inheritance model, was performed using PSEUDOMARKER 2.0^36,37^ based on the haplotype-based haplotype relative risk (HHRR) method and the classical linkage analysis. An additional linkage analysis using a dominant inheritance model was performed to confirm the overlapping peaks seen with the recessive inheritance model. In the second stage, a computationally intensive Pseudomarker analysis was carried out on these candidate markers followed by linkage and association tests. The two-stage method of twostage.py was adopted for the analysis. In line with the previously proposed criteria by Lander and Kruglyak^38^ for linkage signals, we considered evidence of linkage as either significant (logarithm of the odds (LOD) ≥ 3.6; *P* ≤ 2.2 × 10^–5^) or suggestive (LOD ≥ 3.0; *P* ≤ 7.4 × 10^–4^). We tested all four models entailing combinations of with or without linkage versus with or without linkage disequilibrium. The SNPs passing this criterion was annotated for genes, functional consequences, effect prediction and allele frequencies from public databases using Variant Effect Prediction (VEP) tool and interrogated allele frequencies of the SNPs with the Genome Aggregation Database (gnomAD).

**Figure 1:**
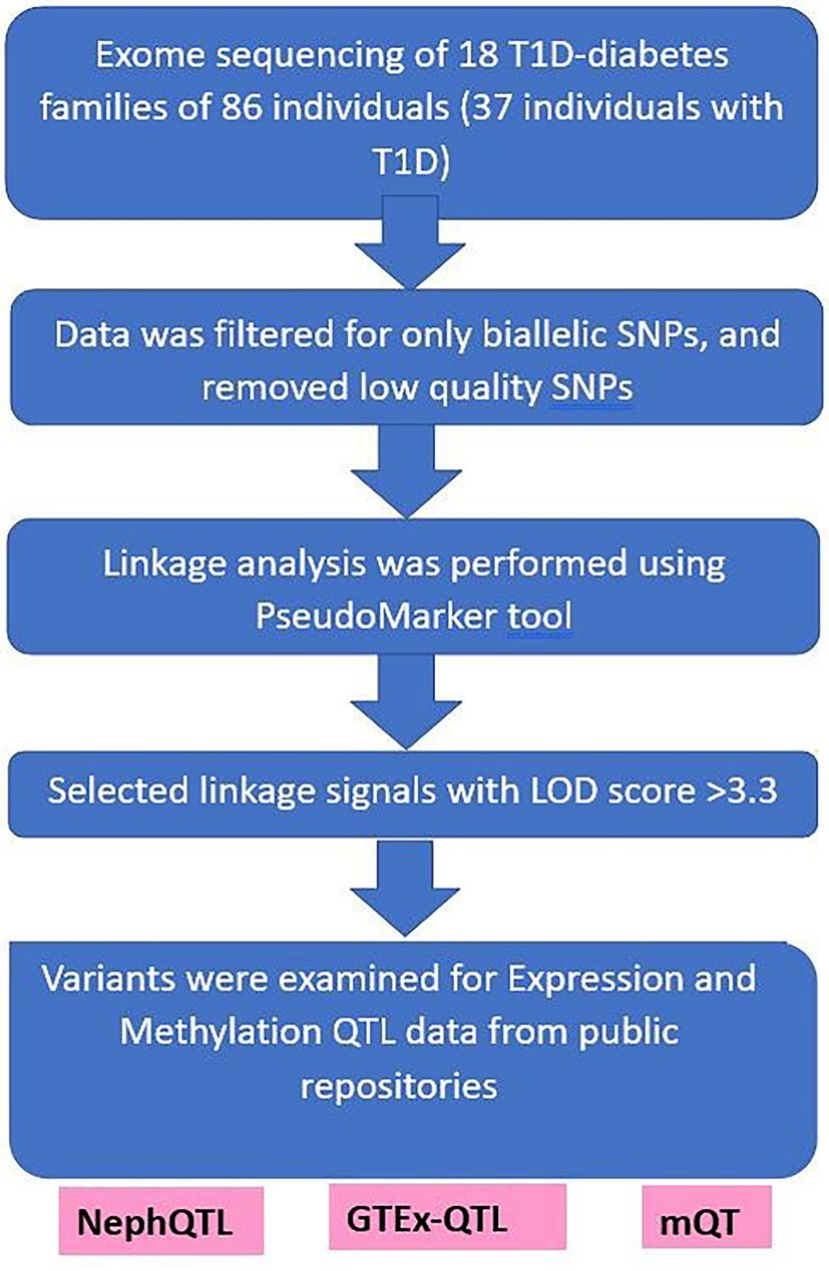
Flowchart of the procedures performed to identify type 1 diabetes-associated linkage signals.

The flowchart of the performed procedures, from exome sequencing to identification of linkage signals, is presented in Fig. 1.

### Building evidence for the linkage signals

The variants with LOD scores of ≥ 3 were investigated for their role in disease etiology by analyzing data on tissue-specific expression of the quantitative trait locus (eQTL) from resources such as the NEPTUNE patient characteristics for subjects in Expression quantitative trait loci (NephQTL)^39^ and genotype-tissue expression (GTEx)-eQTL^40^. Further, by way of utilizing the mQTLdb^41^ (http://www.mqtldb.org/), which is a public resource on lead mQTL loci influencing methylation variation in pregnancy–birth–childhood life courses, we examined the genetic influence of the study variants on differential methylation of CpG sites at different life stages. We examined genome-wide association studies (GWAS) catalog^42^ and literature reports to evaluate the evidence of associations between the lead variants and disease phenotypes. In addition, we investigated the genetic and functional annotation data pertaining to the shortlisted SNPs using Type 1 diabetes Knowledge Portal (T1D-KP)^43^.

### Tag SNP selection and linkage disequilibrium analysis

We shortlisted all Linkage disequilibrium (LD) tag SNPs within *SLC17A1, SLC17A3, TATDN2 and TMEM131L* genes and their 1000 bp flanking regions on both 3’ and 5’ end using TagSNP tool, a modified version of TAGster software from the SNPinfo Web Server (National Institute for Environmental Health Sciences; http://snpinfo.niehs.nih.gov/)^44^. LD was measured by squared correlation (r2) method and the analysis was performed based on dbSNP data. The minimum number of genotype pairs to calculate LD was adopted as 5 and the maximum allowed physical distance between two SNPs for LD calculation was set for 250 KB, and minor allele frequencies were set 0.05–0.5.

### Statistical analysis

Differences in the mean values of plasma glucose and uric acid among individuals with T1D, were calculated using the Mann Whitney test. A *p-*value of < 0.05 is considered as significant.

### Ethics approval and consent to participate

The study was approved by the Ethical Review Committee of Dasman Diabetes Institute and was performed in accordance with the principles of the Declaration of Helsinki, as revised in 2008. Written informed consent was obtained from all study participants. In the case of children, informed consent was obtained from the parents/legal guardians, and an assent was obtained from children aged seven years or older.

## Results

The clinical characteristics of the study participants are detailed in Table 1. Most of the affected people (76%) were children under the age of 18 years; the age at onset of T1D varied from 6 months to 28 years. Approximately 30% of the patients with T1D were overweight. No significant differences in sex distribution were observed among the affected people.

**Table 1:** Clinical characteristics of patients with type 1 diabetes in the study cohort.

Family no	Total no. of family members	No. of affected cases	Relationship^#^	Age (years)	Age at onset (years)	Gender (F/M)	BMI (kg/m^2^)	BP (mmHg)	HbA1C (%)	Uric acid (umol/l)
1	5	3	Daughter	12.6	6	F	26.6	126/69	11	198
			Daughter	12.6	6	F	20.9	122/71	10.2	172
			Son	7.0	5	M	17.0	124/63	10.1	121
2	6	2	Son	13.6	2	M	25.0	124/76	8.4	200
			Daughter	20.0	12	F	18.8	115/70	7	124
3	4	2	Son	8.0	3	M	18.9	109/50	9.3	112
			Son	12.4	9	M	24.4	126/71	8.6	156
4	4	2	Daughter	3.8	2.4	F	16.1	98/77	8.4	
			Mother	26.9	0	F	31.4	118/67	10.2	
5	5	2	Son	14.7	6	M	26.7	128/60	10.3	199
			Son	4.7	4	M	14.4	97/66	8.7	148
6	4	2	Daughter	5.0	4	F	15.1	120/66	11.2	98
			Mother	28.2	28	F	23.9	98/52	8.6	188
7	3	1	Son	16.4	16	M	23.7	118/67	6.1	242
8	5	2	Daughter	17.4	1.8	F	23.2	113/63	8.2	156
			Daughter	15.2	2.2	F	22.9	110/65	11.4	176
9	3	1	Daughter	7.9	6	F	20.5	110/71	11.1	144
10	4	2	Daughter	15.3	10	F	26.8	91/48	7.7	290
			Daughter	10.8	10	F	24.6	86/55	7	249
11	6	2	Son	15.0	2.4	M	18.2	116/63	9.4	230
			Son	20.9	7	M	30.3	112/69	8.2	198
12	5	4	Father	39.3	12	M	25.3	107/63	8.4	249
			Daughter	13.3	11	F	18.5	113/71	8.9	
			Daughter	11.2	10	F	19.1	116/62	7.7	
			Daughter	11.2	10	F	16.8	111/63	9.2	
13	5	2	Daughter	6.6	5	F	14.5	86/56	8.9	
			Father	40.7	13	M	33.3	117/67	8.7	189
14	7	3	Son	25.4		M	14.8	104/58	11.5	133
			Son	24.5		M	17.5	107/74	12.8	202
			Daughter	10.3	0.8	F	15.7	112/65	9.7	190
15	6	2	Son	18.4	6	M	24.7	139/82	10.6	230
			Son	9.5	2	M	16.9	113/68	8.7	183
16	6	1	Son	11.1	10	M	20.8	100/55	8.2	237
17	4	2	Son	5.7	5.7	M	24.5	123/73	8.4	146
			Daughter	8.1	2.8	F	15.2	100/60	10.6	162
18	4	2	Daughter	14.3	1.4	F	24.0	112/62	8.9	187
			Son	9.9	2.6	M	29.9	111/63	8.9	232

F denotes female and M male.

*BMI* body mass index, *BP* blood pressure, *HbA1C* glycated hemoglobin.

^#^Relationship of affected individuals.

### Whole exome based linkage analysis of T1D families

A total of 18 Kuwaiti T1D families consisting of 37 T1D affected individuals and 49 unaffected first-degree relatives were considered. This includes 11 families with sib-pairs concordant for T1D (61%), four families with either a father or mother with T1D (22%) and two families had second degree relatives with T1D (17%). We analyzed exomes of 37 T1D individuals and detected a total of 217,354 single nucleotide variants, of which 46,565 were non-synonymous and 44,513 were synonymous variants. Up to 5% of the detected single nucleotide variants were observed to be novel including personal mutations. A total of 206,203 variants with known rsID were classified as frameshift deletion (n = 382), frameshift insertion (n = 202), non-frameshift deletion (n = 829) and non-frameshift insertion (n = 326). We further detected 118 start loss, 555 stop gain, 60 stop loss and 1 undefined mutation in our cohort.

**Figure 2:**
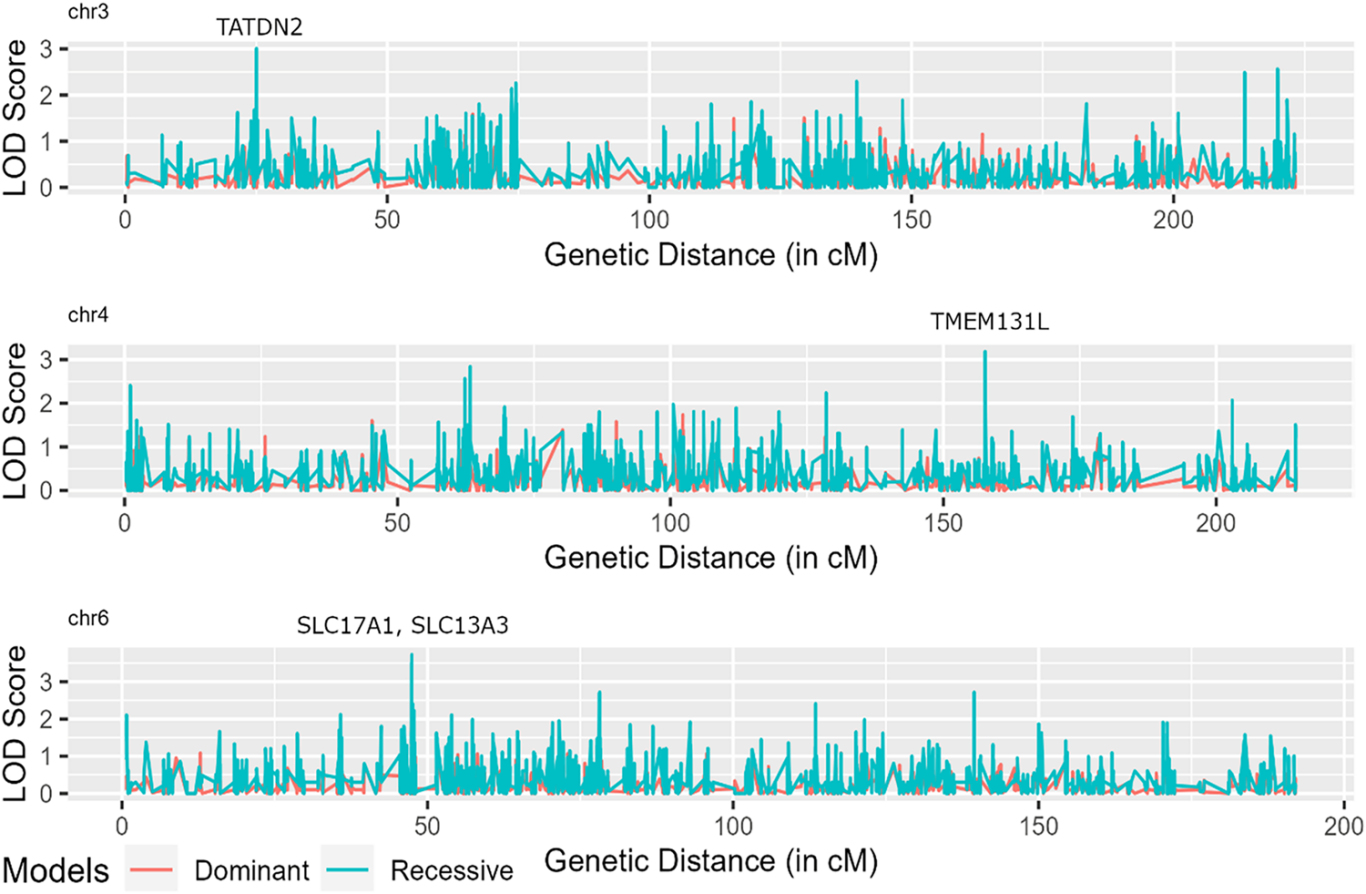
Distribution of the logarithm of the odds (LOD) score across chromosomes 3, 4 and 6.

Five types of linkage analysis tests were performed: tests for linkage with or without allowing for linkage disequilibrium, tests for linkage disequilibrium with or without allowing for linkage, and joint test of linkage and linkage disequilibrium. The analysis pointed to three sites that mapped to chromosomes 3, 4, and 6 (Fig. 2), at which the evidence of linkage under a recessive mode of inheritance was either significant (LOD ≥ 3.6; *P* ≤ 2.2 × 10^–5^) or suggestive (LOD ≥ 3.0; *P* ≤ 7.4 × 10^–4^) (Table 2). The significant linkage signal pointed to the chromosomal region 6p22.2, spanning two genes, namely, *SLC17A1* and *SLC17A3*. The candidate SNPs from this region were rs1165196 (*SLC17A1*:p.T269I) and rs942379 (*SLC17A3*:p.S370S). The two suggestive linkage signals were seen at chromosomal regions 3p25.3 and 4q31.3, harboring *TATDN2* and *TMEM131L*, respectively. The candidate SNPs from these two regions were rs394558 (*TATDN2*:p.V256I) and rs6848033 (*TMEM131L*:p.R190R). These three linkage signals were also seen when the analysis was performed under a dominant mode of inheritance, albeit with no statistical significance.

**Table 2:** Depicts genome-wide linkage signals identified by pseudomarker using recessive and dominant inheritance model.

Region	SNPs	Gene [functional consequences]	Summary statistics of linkage-LD statistical tests^@^			
			Under recessive inheritance model		Under dominant inheritance model	
			Linkage LOD score	*p*-values	Linkage LOD score	*p*-values
6p22.2	rs1165196^$^, rs942379	SLC17A1 [T > I], SLC17A3 [S > S]	3.72	Linkage: 1.7e-05, LD|Linkage: 9.3e-03, LD|NoLinkage:0.09, Linkage|LD: 4.0e-06, LD + Linkage: 4.0e-06	1.75	Linkage: 0.002, LD|Linkage: 0.14, LD|NoLinkage: 0.19, Linkage|LD: 0.003, LD + Linkage: 0.003
3p25.3	rs394558	TATDN2 [V > I]	3.010	Linkage: 9.9e-05, LD|Linkage: 0.09, LD|NoLinkage: 2.3e-02, Linkage|LD: 7.2e-04, LD + Linkage: 1.4e-04	1.12	Linkage: 0.01, LD|Linkage: 0.31, LD|NoLinkage: 0.03, Linkage|LD: 0.19, LD + Linkage: 0.029
4q31.3	rs6848033	TMEM131L [R > R]	3.186	Linkage: 6.4e-05, LD|Linkage: 0.93, LD|NoLinkage: 0.17, Linkage|LD: 3.4e-04, LD + Linkage: 3.8e-04	0.81	Linkage: 0.02, LD|Linkage: 0.014, LD|NoLinkage: 0.13, Linkage|LD: 0.006, LD + Linkage: 0.004

LOD logarithm of the odds. *SLC17A1* solute carrier family 17 member 1, *SLC17A3* solute carrier family 17 member 3, *SNPS* single nucleotide polymorphisms, *TATDN2* tatD DNase domain containing 2, *TMEM131L* transmembrane 131 like.

^@^Linkage: test of linkage without allowing for linkage disequilibrium; LD|Linkage: test of linkage disequilibrium allowing for linkage; LD|NoLinkage: Test of linkage disequilibrium without allowing for linkage; Linkage|LD: Test of linkage allowing for linkage disequilibrium; LD + Linkage: Joint test of linkage and linkage disequilibrium.

^$^GWAS catalog ^31^ reports association of rs1165196 with gout, uric acid concentrations and urinary metabolite concentrations in chronic kidney disease (CKD). PMID: 21983786; PMID: 20884846; PMID: 36281732; PMID: 33832965; PMID: 29403010; PMID: 25811787; PMID: 33356394.

We further investigated additional coding region variants from *TATDN2*, *TMEM131L*, *SLCA17A1* and *SLC17A3 *in our cohort of 18 T1D families consisting of 37 T1D cases and 49 unaffected first-degree relatives (Table 3) based on their enrichment in T1D cohort, their segregation pattern in T1D affected versus unaffected individuals and furthermore based on the supportive evidence from the GTEx, NephQTL and T1D knowledge portal. We tended to prioritize coding region missense variants, since clinical interpretation of the effect of the missense variants is more amenable with missense variants than with non-coding variants. A missense variant from *SLC17A3,* rs56027330, was identified in 6 index cases from 6 T1D families, and in one unaffected relative. These 6 families showed no relatedness as confirmed by examination of kinship using the KING tool. The homozygous alternate allele genotype (TT) at this variant was detected in 6 of 37 T1D children at a frequency of 16.2% (as compared to 1 in 49 unaffected family members), while the 1000 genome phase 3 project reports it as a rare genotype (0.9%). The frequency of this genotype differed considerably across the different ethnicities 0% in AFR, 1.7% in AMR, 0.4% in EAS, 2.4% in EUR, 0.6% in SAS. We additionally detected the homozygous alternate allele genotype (TT) in 3 sporadic T1D cases from another dataset available to us with no family information. When we examined a population-based exome dataset of 291 individuals, with none diagnosed for T1D from Kuwait, only 3.4% (10 individuals) had homozygous alternate TT genotype and 5 of these 10 were diagnosed with T2D.

**Table 3:** Additional missense variants from the candidate genes as seen in 18 T1D families consisting of 37 T1D cases and 49 unaffected first-degree relatives.

Gene/chromosome location/ SNP identifier	Aminoacid change	Global MAF	HomAlt genotypes in T1D family members	HomAlt genotypes in 291 general Kuwaiti population	T1D			Unaffected cases			SIFT	Polyphen
					nHet (0/1)	nHomAlt (1/1)	nHomRef (0/0)	nHet (0/1)	nHomAlt (1/1)	nHom ref (0/0)		
*SLC17A3*/Chr6: 25850845/rs56027330	p.G279R	7% (T)	Confirmed in 6 index cases	10 (3.4%)	3	6 (16.2%)	28	13	1 (2.0%)	35	0.16	0
*TATDN2*/Chr3: 10290952/rs148376271	p.R23H	2% (A)	–	0 (0%)	2	0 (0%)	35	1	0 (0%)	48	0.1	0.802
*TATDN2*/chr3: 10302056/rs2241314	p.H217R	36% (G)	NA	0 (0%)	5	0 (0%)	32	10	1 (2.0%)	38	0.29	0.09
*TATDN2*/chr3: 10302172/rs394558	p.V256I	40% (G)	Confirmed in 5 index cases	97 (33.3%)	17	5 (13.5%)	15	19	2 (4.1%)	28	0.05	0.006
*TATDN2*/chr3: 10311939/rs2075352	p.P358L	18% (T)		5 (0.3%)	5	0 (0%)	32	4	0 (0%)	45	0.01	0.059
*TMEM131L*/Chr4: 154525138/rs36023829	p.T991A	3% (G)	–	0 (0%)	3	0 (0%)	34	2	0 (0%)	47	0.32	0
*TMEM131L*/Chr4: 154,542,906/rs35018723	p.N1254S	2% (G)	–	0 (0%)	1	0 (0%)	36	1	0 (0%)	48	0.39	0.007
*TMEM131L*/Chr4: 154548809/rs35543386	p.A1392P	1% (C)	–	0 (0%)	1	0 (0%)	36	2	0 (0%)	47	0.24	0.893
*TMEM131L*/chr4: 154513627/rs7669418	p.I604V	23% (G)		12 (4.1%)	13	0 (0%)	24	12	2 (4.1%)	35	1	0
*TMEM131L*/chr4:1 54514965/rs17370297	p.M645T	23% (C)		12 (4.1%)	13	0 (0%)	24	13	2 (4.1%)	34	0.21	0.257
*SLC17A1*/chr6:25813150/rs1165196	pT269I	28% (G)	Confirmed in 10 index cases	112 (38.5%)	14	10 (27.0%)	13	16	9 (18.4%)	24	0.26	0.001
*SLC17A3*/chr6:25862466/rs1165165	p.A100T	22% (T)	Confirmed in 2 index cases	24 (8.3%)	10	2 (5.4%)	25	11	3 (6.1%)	35	0.09	0.018

The SIFT score ranges from 0.0 (deleterious) to 1.0 (tolerated); The minimum passing score is 0.4

Genes are italicized.

### Association of lead variants with clinical phenotypes of T1D

**Figure 3:**
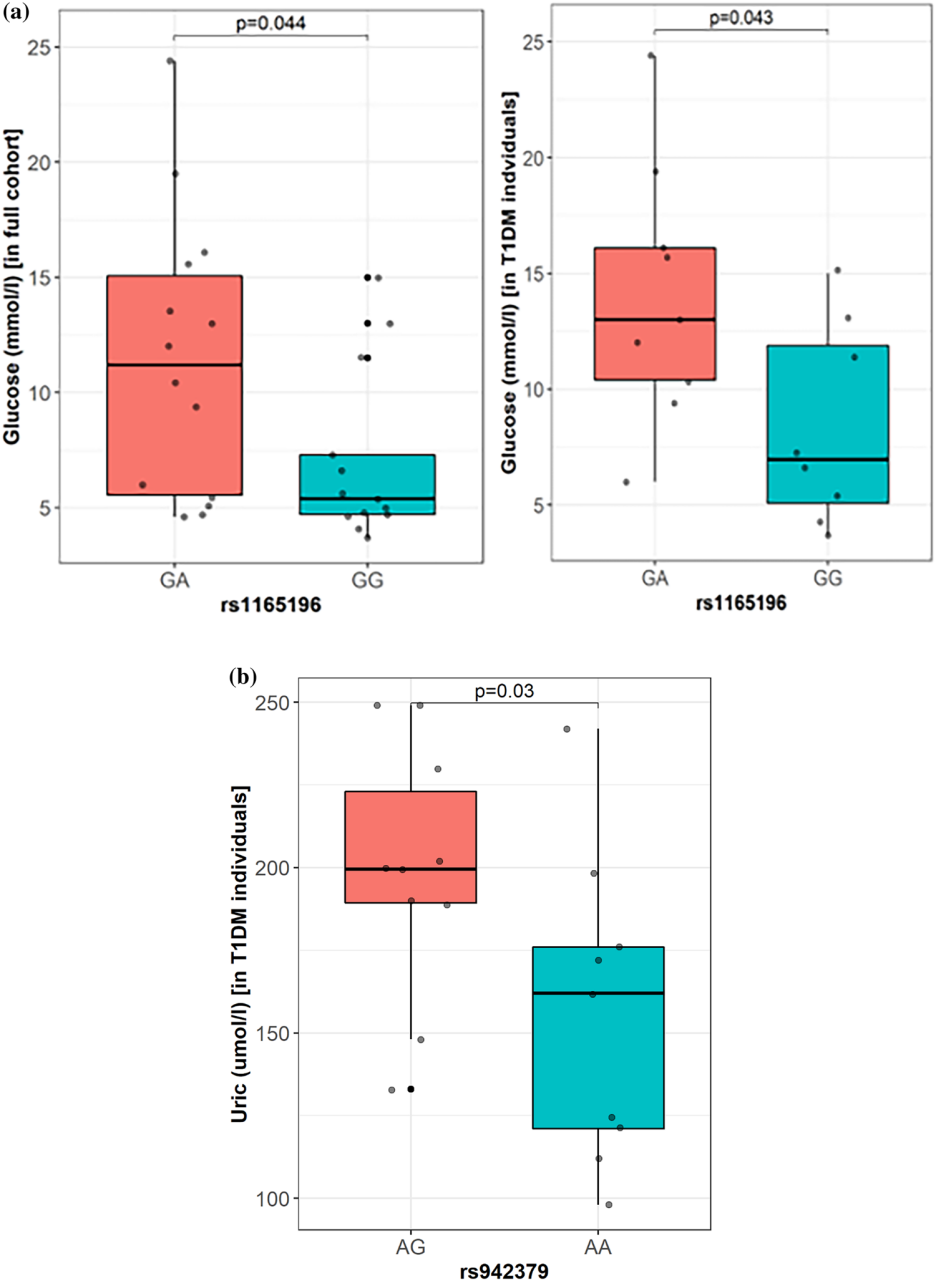
Plasma glucose and uric acid measurements among individuals carrying risk genotypes compared to non-risk genotypes at rs1165196 (risk allele = A; non-risk allele = G) and rs942379 (risk allele = G; non-risk allele = A). (**a**) Differences in the mean values for plasma glucose measurements among the cohort (left panel) or among individuals with type 1 diabetes (right panel) carrying the risk GA genotypes, as compared to those with the non-risk GG genotype at *SLC17A1*:rs1165196. (**b**) Differences in the mean values for uric acid measurements among the type 1 diabetes individuals carrying the risk AG genotype, as compared to those with the non-risk AA genotype at *SLC17A3*:rs942379. There were only 10 non-diabetic participants with available uric acid measurements.

We further investigated the influence of the lead risk variants on the phenotypic traits relating to the study participants. A statistically significant increase in plasma glucose measurements was observed among individuals with T1D carrying the *SLC17A1* rs1165196 GA genotype, as compared to those with the GG genotype (*p* = 0.043) (Fig. 3a). Irrespective of their diabetic status, individuals carrying the GA genotype showed a significantly higher plasma glucose measurement, as compared to those carrying the GG genotype (*p* = 0.044). Similarly, patients with T1D carrying the *SLC17A3* rs942379 AG genotype showed significantly higher uric acid concentration compared with those with the GG genotype (*P* = 0.03) (Fig. 3b). As there were only 10 non-diabetic participants with available data on uric acid measurements, analysis with the combined cohort was not performed. The other two linked variants did not show any statistically significant impact on the clinical profiles of the patients with T1D.

### Lead variants and quantitative trait loci (QTL) analysis

**Figure 4:**
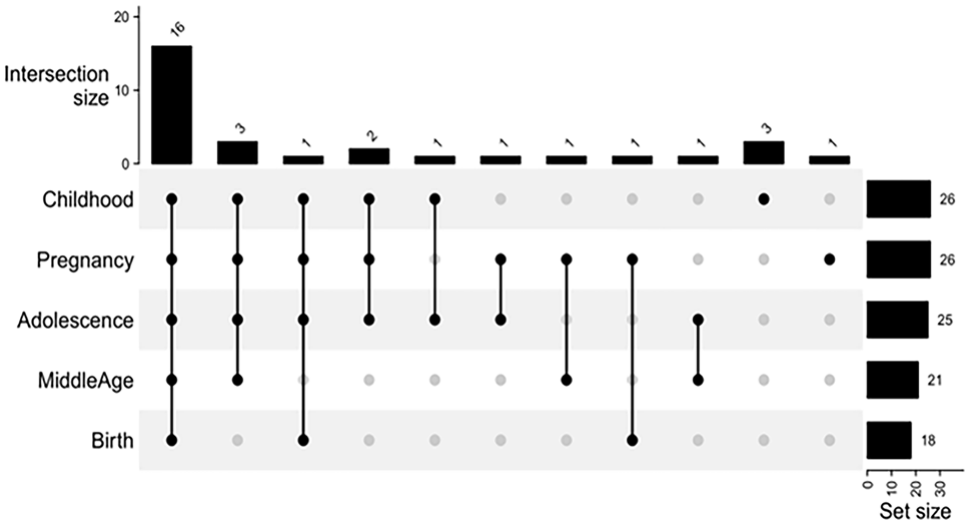
An UpSet plot illustrating cross-comparison of CpG sites across various life stages. The line joining the black dots indicates shared CpGs in the stages of life, while the vertical bar shows number of CpGs shared between the stages of life. The horizontal bar indicates the number of CpGs in each life stage.

**Figure 5:**
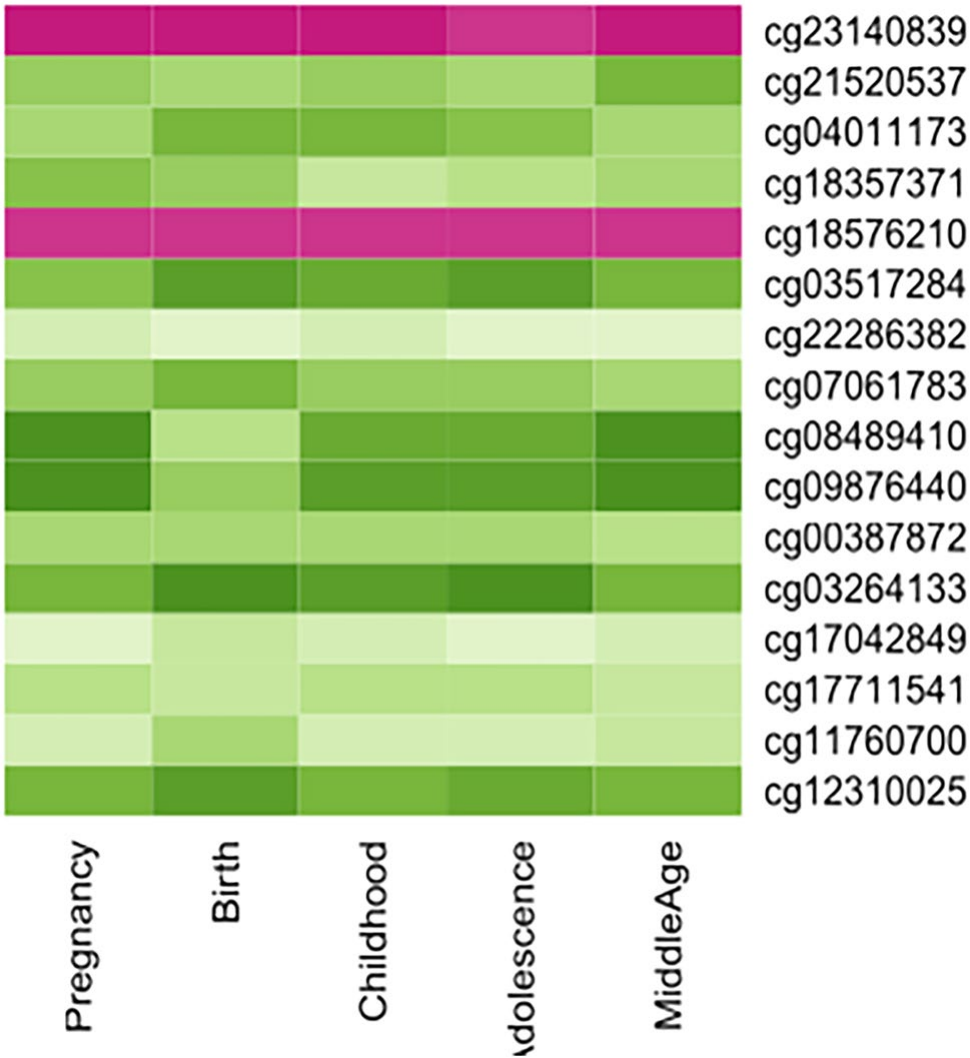
Shared CpGs across different life stages. Consistent methylation direction in 16 shared CpG sites across the life stages, suggesting that these shared CpGs have no contribution to childhood type 1 diabetes.

Further, examination of the mQTLdb revealed that the lead linkage variants *SLC17A1* rs1165196, *SLC17A3* rs942379, and *TATDN2* rs394558 had genetic influence on DNA methylation at various CpG sites in different stages of life (Supplementary Table S1). Depending on the genotypes at the above-mentioned three SNPs, we found 26 differentially methylated CpG sites at the life stage of pregnancy, 18 sites at birth, 26 sites at childhood, 25 sites at adolescence, and 21 sites at middle-age (Fig. 4). A cross-comparison of these CpG sites across all the life stages found that 16 of them were common across all life stages, three sites were common between pregnancy–childhood–adolescence–middle age, one between pregnancy–birth–childhood–adolescence, two between pregnancy–childhood–adolescence, and one each between childhood–adolescence, adolescence–pregnancy, pregnancy–middle age, and pregnancy–birth. Furthermore, one unique CpG site in pregnancy and three unique CpG sites in childhood were seen. The direction of methylation among the 16 shared CpG sites across the different life stages showed no significant variation among the tested individuals (Fig. 5). The three CpG sites (Table 4) that were seen as unique to the childhood stage could be the key signatures mediating phenotypic changes in children. Two of these three CpG sites, namely cg17978425 (Beta = − 0.30; *P* = 1.40E-12) and cg10346111 (Beta = 0.25; *P* = 1.95E-09), are both regulated by *TATDN2* rs394558. However, they differed in the direction of methylation with the former CpG site being hypo-methylated and the latter hyper-methylated (see Table 4). Further, the prioritized missense variant *SLC17A3* rs56027330 also had genetic influence on DNA methylation at five CpG sites in different stages of life (see Supplementary Table S1); all these five sites are also influenced by the linked lead variants. One of these five CpG sites, namely cg12310025, is common across the five life stages of adolescence, birth, childhood, middle age, and pregnancy; two of these five sites, namely cg03264133 and cg03517284, are common across four of the above five life stages; two other CpG, namely cg23140839 and cg07061783, are seen in only one of the life stages.

**Table 4:** mQTL statistics of CpGs regulated by rs394558 and rs942379 found to be unique in childhood.

SNP	SNP location	Alleles	CpG	CpG location	Beta	*t* stat	*p*-value	Functional consequences of methylation site
rs394558	3:10302172	A/G	cg17978425	3:10,289,658	− 0.30	− 7.19	1.40E-12	Downstream of *TATDN2*
rs394558	3:10302172	A/G	cg10346111	3:10,326,661	0.25	6.07	1.95E-09	Upstream of *TATDN2* and *GHRL,* overlaps on *LINC00852* and intronic region of *GHRLOS*
rs942379	6:25849620	A/G	cg12588279	6:26,043,732	− 0.19	− 5.41	8.12E-08	*H2BC3/HIST1H2BB*

*GHRL* ghrelin and obestatin prepropeptide, *GHRLOS* ghrelin opposite strand/antisense RNA, *H2BC3/HIST1H2BB* H2B clustered histone 3, *LINC00852* long intergenic non-protein coding RNA 852, *SNP* single nucleotide polymorphism, *TATDN2* tatD DNase domain containing 2.

Examination of the NephQTL database to determine which of the lead variants act as eQTL in kidney revealed that the *SLC17A1* rs1165196, *SLC17A3* rs942379, rs56027330, and *TATDN2* rs394558 exhibited genotype-based differential expression of genes in either glomerular or tubular tissues (Table 5). Similarly, the GTEx database depicted rs942379 and rs394558 as eQTL variants that influence the expression of certain key genes in the pancreas and testes (Table 6). The prioritized *SLC17A3* rs56027330 variant also tends to significantly impact the expression of several key genes such as *SLC17A1, SLC17A3,* and *TRIM38* in multiple tissues indicating its pleiotropic effect (Table 7).

**Table 5:** Variants identified as eQTL with kidney tissue-specific expression from the NephQTL database.

Linkage SNP observed in our study (Alleles)	Differentially expressed gene^@^	Kidney tissue exhibiting differential expression	Alternate allele frequency^$^	Beta*	*t*-statistics^&^	*p-*value^#^
rs1165196 (G/A)	*SLC17A4*	Tubulointerstitium	0.68	0.29	4.16	5.67E-05
rs1165196 (G/A)	*HIST1H2BA*	Tubulointerstitium	0.68	0.27	2.65	9.15E-03
rs1165196 (G/A)	*HIST1H2AA*	Tubulointerstitium	0.68	0.28	2.16	3.23E-02
rs1165196 (G/A)	*SLC17A3*	Tubulointerstitium	0.68	0.16	2.09	3.82E-02
rs1165196 (G/A)	*HIST1H4F*	Tubulointerstitium	0.68	− 0.22	− 2.03	4.42E-02
rs1165196 (G/A)	*HIST1H1A*	Tubulointerstitium	0.68	− 0.20	− 2.03	4.48E-02
rs942379 (A/G)	*HIST1H2BE*	Glomerulus	0.66	0.40	2.5	0.015
rs942379 (A/G)	*SLC17A4*	Tubulointerstitium	0.66	0.26	3.77	2.42E-04
rs942379 (A/G)	*HIST1H2BA*	Tubulointerstitium	0.66	0.31	3.07	2.59E-03
rs942379 (A/G)	*SLC17A3*	Tubulointerstitium	0.66	0.18	2.34	2.07E-02
rs942379 (A/G)	*HIST1H4F*	Tubulointerstitium	0.66	− 0.23	− 2.16	3.27E-02
rs942379 (A/G)	*HIST1H2AA*	Tubulointerstitium	0.66	0.25	2.01	4.70E-02
rs394558 (G/A)	*ATP2B2*	Glomerulus	0.46	− 0.37	− 3.19	1.94E-03
rs56027330 (C/T)	*SLC17A3*	Glomerulus	0.07	0.40	2.0	0.047

*ATP2B2* ATPase plasma membrane Ca2 + transporting 2, *eQTL* expression of the quantitative trait locus, *HIST1H1A* H1.1 linker histone, cluster member, *HIST1H2AA* H2A clustered histone 1, *HIST1H2BA* H2B clustered histone 1, *HIST1H2BE* H2B clustered histone 6, *HIST1H4F* H4 clustered histone 6, *NephQTL* NEPTUNE patient characteristics for subjects in Expression quantitative trait loci, *SLC17A3* solute carrier family 17 member 3, *SLC17A4* solute carrier family 17 member 4, *SNP* single nucleotide polymorphism.

^@^Genes that are differentially expressed depending on the genotype seen at the variant from the linkage signal in the cohort of individuals with either glomerular or tubulointerstitium expression data in NephQTL database.

^$^The alternative allele frequency in subjects with either glomerular or tubulointerstitium expression data and genetic data.

*Estimated regression coefficient from MatrixEQTL.

^&^Beta coefficient divided by estimated standard error.

^**#**^p-value corresponds to the probability of obtaining the observed t-statistic.

**Table 6:** Variants identified as QTL from the GTEx portal.

Linkage SNP observed in our study	Differentially regulated genes	Tissue	Normalized effect size	*p*-value
rs394558	*IRAK2*^*#*^	Adipose-subcutaneous	− 0.27	4.80E-08
rs394558	*TATDN2*	Pancreas	0.31	1.60E-12
rs394558	*GHRLOS*	Pancreas	0.44	4.40E-12
rs394558	*LINC00852*	Pancreas	0.33	0.000001
rs394558	*LINC00852*	Testis	0.5	1.10E-11
rs942379	*TRIM38*^*#*^	Colon	0.4	3.40E-07
rs942379	*TRIM38*	Pancreas	− 0.24	0.0000016
rs942379	*SLC17A3*	Testis	0.51	9.20E-12
rs942379	*HIST1H2APS5*	Testis	0.33	1.00E-08
rs942379	*HIST1H2BA*	Testis	− 0.36	2.10E-08
rs942379	*TRIM38*	Testis	− 0.1	0.00013
rs1165196	*TRIM38*^*#*^	Colon	0.4	1.20E-07
rs1165196	*SLC17A3*	Testis	0.51	8.70E-12
rs1165196	*HIST1H2BA*	Testis	− 0.37	3.90E-09
rs1165196	*HIST1H2APS5*	Testis	0.29	7.10E-07

*IRAK2* Interleukin-1 receptor associated Kinase2, *GHRLOS* ghrelin opposite strand/antisense RNA, *GTEx* genotype-tissue expression, *HIST1H2APS5* H2A clustered histone 5, pseudogene, *HIST1H2BA* H2B clustered histone 1, *LINC00852* long intergenic non-protein coding RNA 852, *SLC17A3* solute carrier family 17 member 3, *SNP* single nucleotide polymorphism, *TATDN2* tatD DNase domain containing 2, *TRIM38* tripartite motif containing 38.

^#^sQTL stands for splicing quantitative trait loci. The rest of the QTL shown in the table are eQTL, expression of the quantitative trait locus.

**Table 7:** Identified eQTL related to rs56027330 variant from the GTEx portal.

Gene symbol	p-value	NES	Tissue
*SLC17A1*	1.90E-24	1.3	Adrenal gland
*SLC17A3*	2.60E-12	0.88	Adrenal gland
*SLC17A1*	1.10E-10	0.68	Liver
*TRIM38*	1.40E-07	− 0.25	Thyroid
*SLC17A3*	0.000001	0.43	Liver
*SLC17A3*	0.0000022	0.67	Kidney—cortex
*SLC17A3*	0.0000044	0.53	Testis
*HIST1H3E*	0.000043	0.35	Testis
*HIST1H3E*	0.000069	0.27	Whole blood

*eQTL* expression of the quantitative trait locus, *SLC17A1* solute carrier family 17 member 1, *SLC17A3* solute carrier family 17 member 3, *TRIM38* tripartite motif containing 38, *HIST1H3E* histone cluster 1 H3 Family Member E.

### Functional annotations of lead variants based on T1D knowledge portal

Supportive evidence from the T1D Knowledge portal indicates significant association of the lead variant from *SLC17A1* (rs1165196) and *SLC17A3* (rs942379) with T1D, HbA1C, serum urate, and uric acid. *TATDN2* rs394558 variant also showed significant association with HbA1C, Matsuda insulin sensitivity index and several other insulin related parameters as shown in (Table 8). Similarly, *TMEM131L* rs6848033 variant presented evidence for significant association with Matsuda insulin sensitivity index (ISI), fasting insulin, HOMAIR, and HOMAB. Additionally, the missense variant rs56027330 shortlisted by our study consistently showed association with T1D, HbA1C, serum urate and diabetic nephropathy alongside several other nephropathy-related traits such as creatinine, sodium excretion, and mean corpuscular hemoglobin.

**Table 8:** Phenome wide associations related to lead variants from the Type 1 diabetes knowledge portal.

Variants	Phenotype	P-value	Beta
rs1165196	HbA1C	9.38E-15	− 0.008
	HbA1C adjusted BMI	0.0003	− 0.0123
	Serum urate	5E-324	0.0718
	Uric acid	1.43E-19	0.0516
	T1D	7.58E-12	0.0893
rs942379	HbA1C	4.06E-20	− 0.0088
	HbA1C adjusted BMI	5.00E-05	− 0.0137
	Serum urate	5E-324	0.0685
	Uric acid	1.01E-17	0.0486
	T1D	1.94E-14	0.0978
rs394558	Lymphocyte count	0.0006845	0.0057
	Monocyte count	0.000732	0.0054
	Incremental insulin at 30 min OGTT	0.008891	0.057
	Insulin at 30 min OGTT	0.01758	0.052
	Insulin at 30 min OGTT adj BMI	0.02551	0.049
	Area under the curve (AUC) for insulin	0.03721	0.046
	Latent autoimmune diabetes in adults	0.03891	0.0788
	Urinary potassium-to-creatinine ratio	0.04624	0.0047
	AUC insulin over AUC glucose	0.05075	0.044
	HbA1C	0.05155	0.0017
	Matsuda insulin sensitivity index (ISI)	0.05246	− 0.043
rs6848033	Mean platelet volume	1.73E-07	− 0.0089
	Matsuda insulin sensitivity index (ISI)	0.001632	− 0.084
	Fasting insulin	0.01411	− 0.0078
	HOMAIR	0.02111	− 0.0098
	HOMAB	0.02684	− 0.0079
	AUC for insulin	0.03281	0.056
	Fasting glucose adjusted BMI	0.04152	− 0.0032
	Urinary sodium-creatinine ratio	0.04511	− 0.005
	Urinary potassium-to-creatinine ratio	0.04933	− 0.0047
	Insulinogenic index	0.04951	− 0.128
rs56027330	Mean corpuscular hemoglobin	8.62E-292	0.1037
	Serum urate	5.60E-114	0.0571
	Hemoglobin conc	1.85E-73	0.0441
	HbA1C	1.77E-52	− 0.0212
	HbA1C adjusted BMI	2.51E-13	− 0.0345
	eGFRcrea (serum creatinine)	1.10E-27	0.01957
	Creatinine	2.32E-21	− 0.0229
	Monocyte count	7.76E-08	0.0158
	Cystatin C	5.07E-07	− 0.0135
	Na excretion	0.000014	0.0084
	Serum urea	0.0002809	0.0107
	T1D	0.01628	− 0.0384
	Diabetic nephropathy	0.0283	− 0.0684

### Tag SNP and QTL associations

As an indirect approach, we captured the extended list of variants that are in linkage disequilibrium (LD) with the lead variants by haplotype-tagging method. Notably, the lead variants from *SLC17A1* rs1165196 and *SLC17A3* rs942379 are completely in LD with each other. The schematic diagram of all tag SNPs and their pattern of Linkage disequilibrium (LD) is shown in Supplementary Fig. S3. *SLC17A1* rs1165196 represented a haplotype block consisting of 23 SNPS, while *SLC17A3* rs942379 and *TATDN2* rs394558 captured 4 variants each in LD, while *TMEM131L* rs6848033 variant was observed in singleton LD.

We investigated the allelic effect of the captured tag SNPs using mQTLdb and NephQTL databases. Tag SNPs captured by *SLC17A1* rs1165196 variant tends to profoundly impact 1752 CpG sites, while those captured by *SLC17A3* rs942379 variant are likely to influence 337 CpG site. Likewise, the tag SNPs captured by *TATDN2* rs394558 variant significantly associates with 126 CpG sites across different life stages.

Evidence from the NephQTL database indicates that tag SNPs associated with *SLC17A1* rs1165196 and *SLC17A3* rs942379 variants collectively tend to impact the expression of *HIST1H2BE* in glomerulus. Tag-SNPs captured by *SLC17A3* rs942379 variant additionally impacts the expression of *BTN3A2* in glomerulus and those captured by *TATDN2* rs394558 variant combinedly affects the expression of *ATP2B2* in glomerulus further elucidating its role in nephropathy related traits.

## Discussion

Variation in the risk of developing T1D can largely be attributed to genetic predisposition, gene–environment interactions, and other prevailing factors such as ethnic disparities and rapid changes in lifestyle and dietary practices. It is challenging to identify the unique risk variants that segregate in the affected members of multiple families. In the present study, we aimed at minimizing heterogeneity and confounding factors by adopting a family-based linkage and fine-mapping approach to pinpoint genetic markers underlying T1D in the Kuwaiti population. We identified three potential loci with a LOD score of ≥ 3, spanning four candidate genes, namely *SLC17A1, SLC17A3, TATDN2,* and *TMEM131L* in Kuwaiti families diagnosed with T1D. The identified lead linkage variants (*SLC17A1-*rs1165196: pT269I*, SLC17A3*-rs942379: p.S370S*, TATDN2*-rs394558:p.V256I, and *TMEM131L-*rs6848033:p.R190R and the missense variant with the genotype homozygous for the alternate allele (*SLC17A3* rs56027330_p.G279R) enrichening the affected members of Kuwaiti T1D families have not been previously linked with T1D and could be novel contributions to the existing literature.

Though the *SLC17A3* rs56027330 missense variant is a common variant in the 1000 Genomes Phase3 Project samples at 7% MAF, the genotype homozygous for the alternate allele (TT) is rare at 0.9% in the 1000 Genomes Phase 3 Project samples; the frequency differed considerably among the continental populations—2.4% in EUR, 1.7% in AMR, 0.6% in SAS, 0.4% in EAS, and 0% in AFR. It is interesting to note that this genotype is seen in 16.2% of the T1D individuals in our study cohort. Furthermore, we observe this genotype in 3 T1D individuals from another dataset available to us with no family information. In the population-based exome dataset of 291 individuals, with none diagnosed for T1D from Kuwait, only 10 individuals (3.4%) had the homozygous alternate genotype and 5 of these 10 were diagnosed with T2D. The SIFT score (0.0 (deleterious) to 1.0 (tolerated) for this variant is 0.16 much lower than the minimum passing score of 0.4. Existing evidence from T1D Knowledge Portal, linking rs56027330 variant to T1D on GWAS meta-analysis of European ancestry, further substantiates the significance of the shortlisted variant in T1D etiology^43^. It is also the case that the T1D Knowledge Portal provides evidence for association of the variant with HbA1C trait in data sets including the UK Biobank on European ancestry, and the AMP T2DKP on European, East Asian and trans-ancestry. The observed recessive model of association for the rs56027330 variant with T1D in our study cohort is important given the accumulation of recessive alleles in the Arab population, whose genetic profile has evolved through practice of consanguinity and the resultant inbreeding^45,46^. Given the small size of the study cohort, it is recommended that further replication studies are performed in future to unravel the homozygous effect of rs56027330 variant on T1D and glycemic traits.

The top priority lead linkage signals from chromosome 6p22.2 revealed two candidate genes—*SLC17A1* and *SLC17A3*. Both genes are primarily involved in urate metabolism and transport^47,48^. Given that 50% of patients with T1D are prone to developing diabetic kidney disease and that serum uric acid may be a modifiable risk factor of nephropathy in T1D^49–51^, the identification of a genetic variant that affects urate concentration is of high clinical significance, especially that high uric acid level has been implicated in β-cell dysfunction^52^ Our study can potentially contribute to the predictive roles of *SCL17A1* rs1165196 and *SLC17A3* rs942379 variants in the early diagnosis and prediction of T1D and its complications, particularly diabetic kidney disease. In agreement with this, large-scale GWAS have specifically associated the *SCL17A1* rs1165196 variant with uric acid concentration in both European^53–55^ and Japanese individuals^56^, and with gout in those with a European background^57^. Other SNPs from *SLC17A3* are also known to be associated with uric acid concentrations in European^58^ and Korean individuals^59^, and with urinary metabolite measurements in Europeans with chronic kidney disorder^60^. Moreover, *SLC17A3* has been linked to gout susceptibility^61^. A recent study has shown a significant effect of the *SLC17A3* rs942379 variant on the longitudinal measurement of uric acid in women^58^. The NephQTL database additionally indicates the influential role of *SCL17A1* rs1165196 and *SLC17A3* rs942379 variants in regulating allele-specific expression of specific histone and solute carrier genes in kidney tissues, and this observation further indicates these variants as potential markers for progression to renal disease in T1D. Likewise, *SLC17A3* rs942379, which is a synonymous variant, seems to regulate *HIST1H2BB *via the mQTL for cg12588279 CpG site.

The prioritized missense variant *SLC17A3* rs56027330 also had genetic influence on DNA methylation at five CpG sites in different stages of life. The variant also tends to significantly impact the expression of several key genes such as *SLC17A1, SLC17A3,* and *TRIM38* in multiple tissues. Currently, there is no functional evidence linking *SCL17A1* and *SLC17A3* to T1D etiology. Evidence obtained from single tissue QTL analysis indicates the allelic effect of *SLC17A3* rs942379 on the expression of *TRIM38* in colon and pancreatic tissues, while *SLC17A1* rs1165196 tends to be associated only with the expression *TRIM38* in colon tissues. Given the fact that *TRIM38* clusters within major histocompatibility region-1 and caters multiple innate immune and inflammatory responses^62,63^ its direct association with the linked variants may suggest a common pathogenesis that needs to be further validated.

The two additional loci detected in our study include chromosome 3p25.3 and 4q31.3 regions encoding KIAA genes, namely *TATDN2* (KIAA0218) and *TMEM131L* (KIAA0922), respectively. *TATDN2* is predominantly involved in metal ion binding and has been largely related to the unfolded protein response pathway, which in turn plays a crucial role in alleviating endoplasmic reticulum stress by facilitating the degradation of misfolded proteins^64^. A defective unfolded protein response has been proposed to contribute to β-cell apoptosis, leading to T1D^64,65^. *TMEM13L* is negatively involved in regulating the Wnt signaling and thymocyte proliferation pathways. The canonical Wnt signaling pathway has been widely implicated in various renal diseases, such as diabetic nephropathy, fibrosis, renal cancer, and renal failure^66–68^.

The functional roles of *TATDN2* rs394558 and *TMEM131L* rs6848033 are yet to be well-characterized. The NephQTL database shows that the *TATDN2* rs394558 variant tends to significantly impact the expression of *ATP2B2* in glomerulus tissue, indicating its potential role in renal function. *TATDN2* rs394558 variant also significantly influences the expression profile of several key genes such as *IRAK2* in subcutaneous adipose tissue and *TATDN2*, *GHRLOS,* and *LINC00852* in pancreatic tissues. *IRAK2* is a member of serine-threonine kinase family that plays a critical role in regulating inflammatory responses^69,70^, by mediating interleukin-1 induced activation of NF-Kappa-B signaling (PMID 21495968). *TATDN2* rs394558 is also predicted to be involved in regulating genes such as *TATDN2, GHRL, GHRLOS,* and *LINC00852, *via mQTL for cg10346111 CpG sites. The variant may engage in crosstalk with DNA methylation, and thereby impact the transcription of these genes or their nearby genes.

Interestingly, *TATDN2* rs394558 variant tends to impact the expression of both *GHRL* and its antisense gene *GHRLOS*. Functionally, *GHRL* encodes the ghrelin–obestatin preproprotein that is cleaved to yield ghrelin and obestatin. Ghrelin is a powerful appetite stimulant that plays a vital role in energy homeostasis and pancreatic glucose-stimulated insulin secretion, while obestatin is involved in multiple metabolic events, including adipocyte function and glucose metabolism^71^. *GHRLOS* (ghrelin opposite strand/antisense RNA) is an antisense transcript for the *GHRL* gene that may potentially regulate the expression of ghrelin–obestatin preproprotein. The circulating levels of *GHRLOS* and *LINC00852* are reported to be elevated in type 2 diabetes mellitus^72^, while its precise role in T1D remains to be explored.

Our study highlights genes linked to T1D and their prospective role in disease etiology by regulating the expression of genes implicated in inflammatory and metabolic events, as evidenced by publicly available QTL data. Further replicative and longitudinal studies are needed in the future to validate the associations for these loci obtained in the present study.

## Conclusion

Very few studies have explored the genetic epidemiology of T1D in Arab populations^19,32^. The present study suggests *SLC17A1*, *SLC17A3*, *TATDN2,* and *TMEM131L* as candidate genes linked to T1D in Kuwaiti families. The supportive evidence obtained from mQTLdb, NephQTL, GTEx and T1D KP databases provides a suggestive role for *SCL17A1* and *SLC17A3* in T1D susceptibility. The study further highlights the *SLC17A3* rs56027330_p.G279R variant as a potential marker for T1D; the variant associates with T1D under the genetic model for recessive mode of inheritance. This observation is interesting given the accumulation of recessive alleles in the Arab population, whose genetic profile has evolved through practices of consanguinity and the resultant inbreeding. Thus, this variant deserves further follow-up. A major limitation is the small size of the study cohort and the lack of an independent cohort for replicating the findings. It is hoped that future studies from the region aim at replicating these findings in a larger independent T1D cohort and aim at delineating the mechanistic details of the impact of these novel candidate genes on the pathophysiology of T1D.

### Supplementary Information

Supplementary Legends.

Supplementary Figure S1.

Supplementary Figure S2.

Supplementary Figure S3.

Supplementary Table S1.

## Data Availability

The datasets used and/or analyzed during the current study are available from the corresponding authors upon reasonable request.
