# Supplementary material for: Linkage analysis using whole exome sequencing data implicates SLC17A1, SLC17A3, TATDN2 and TMEM131L in type 1 diabetes in Kuwaiti families
Source: Sci Rep. 2023 Sep 11;13:14978. doi: 10.1038/s41598-023-42255-2 (PMC10495342; doi:10.1038/s41598-023-42255-2)
Supplement: Supplementary file 1 — Supplementary Legends. [file 41598_2023_42255_MOESM1_ESM.docx]

**List of Supplementary Tables**

**Supplementary Table S1:** mQTL associated with *SLC17A1* rs1165196, *SLC17A3* rs942379, and *TATDN2* rs394558 in blood during various stages of life.

**List of Supplementary Figures**

**Supplementary Fig. S1:** Whole exome sequencing protocol using Nextera Rapid capture Exome kit (Illumina, USA).

**Supplementary Fig. S2:** Represents extended pedigree of families shortlisted for the study. Pink dot indicates individuals affected with Type 1 diabetes (T1D, and blue those affected with Type 2 diabetes. Arrow indicates index case.

**Supplementary Fig. S3:** Linkage Disequilibrium plot indicating the tag SNPs captured within *SLC17A1,* *SLC17A3, TATDN2* and *TMEM131L* genes.
