# Supplementary figures and images for: Linkage analysis using whole exome sequencing data implicates SLC17A1, SLC17A3, TATDN2 and TMEM131L in type 1 diabetes in Kuwaiti families
Source: Sci Rep. 2023 Sep 11;13:14978. doi: 10.1038/s41598-023-42255-2 (PMC10495342; doi:10.1038/s41598-023-42255-2)

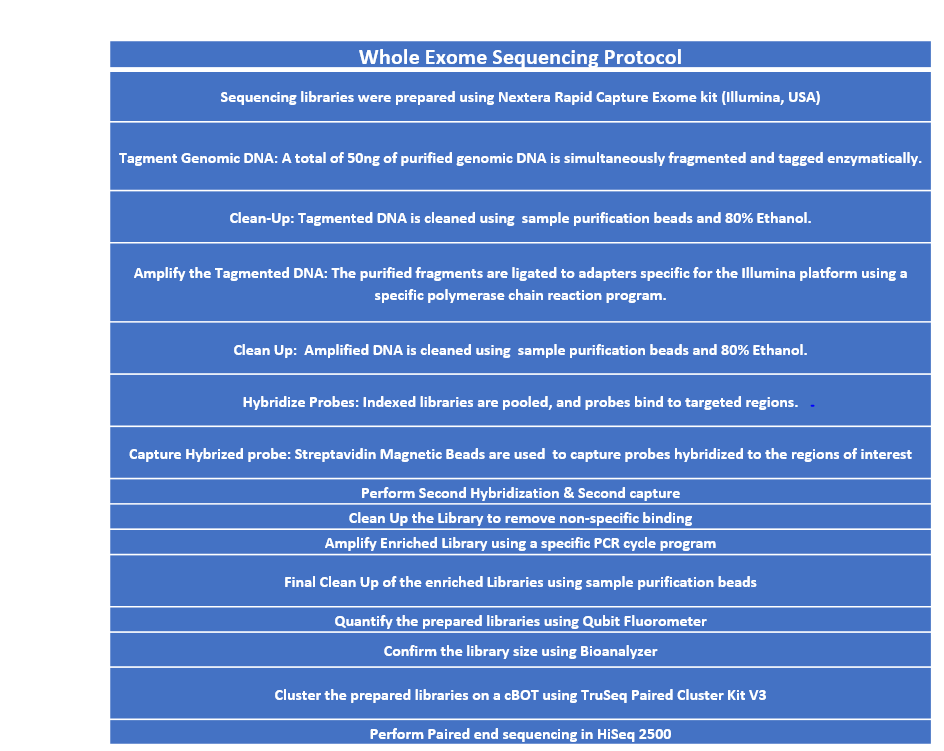

Supplement: Supplementary file 2 — Supplementary Figure S1. [file 41598_2023_42255_MOESM2_ESM.tif]

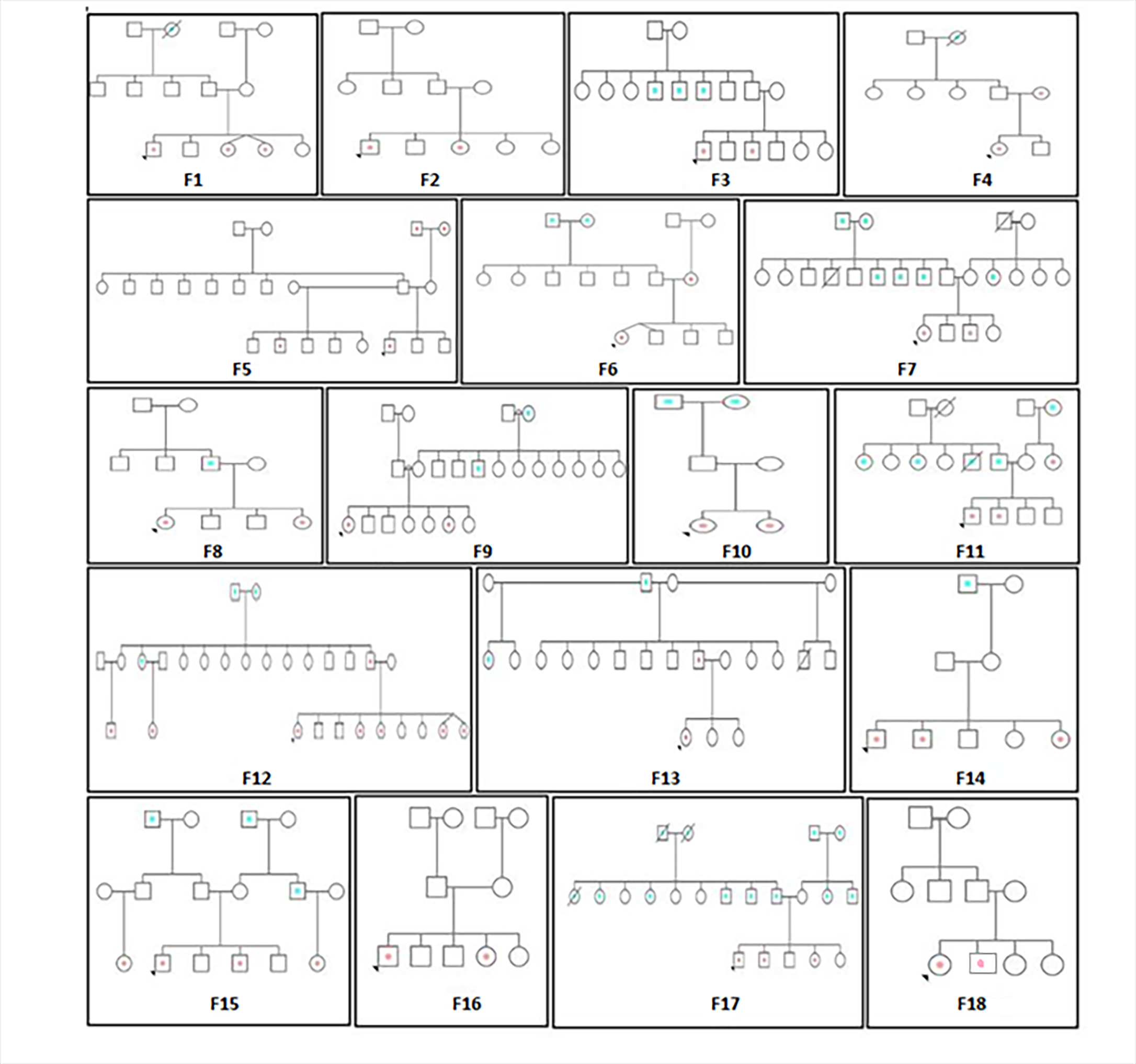

Supplement: Supplementary file 3 — Supplementary Figure S2. [file 41598_2023_42255_MOESM3_ESM.tif]
